# Data from X-ray crystallographic analysis and DFT calculations on isomeric azo disperse dyes

**DOI:** 10.1016/j.dib.2018.10.010

**Published:** 2018-10-09

**Authors:** Jihye Lim, Malgorzata Szymczyk, Nahid Mehraban, Yi Ding, Lisa Parrillo-Chapman, Ahmed El-Shafei, Harold S. Freeman

**Affiliations:** College of Textiles, North Carolina State University, Raleigh, NC 27606, USA

## Abstract

X-ray crystallography and DFT calculations were used to characterize the molecular nature and excited state properties of isomeric photostable azo dyes for textile fibers undergoing extensive sunlight exposure. Structural data in CIF files arising from X-ray analysis are reported and the complete files are deposited with the Cambridge Crystallographic Data Centre as CCDC 1548989 (https://www.ccdc.cam.ac.uk/structures/Search?Ccdcid=1548989) and CCDC 1548990 (https://www.ccdc.cam.ac.uk/structures/Search?Ccdcid=1548990). Data from calculating the vertical electronic excitation of 20 excited states for each dye and from calculating excited state oxidation potential (ESOP) and Frontier HOMO/LUMO isosurfaces are also presented. This data is related to the article “Molecular and excited state properties of isomeric scarlet disperse dyes” (Lim et al., 2018) [1].

## Specifications table

**Table:** 

Subject area	Chemistry, Photophysics
More specific subject area	Inkjet printing, Azo Dyes, Excited State Properties, X-ray Crystallography.
Type of data	Table, Image (x-ray, TD-DFT calculations), Figure
How data was acquired	X-ray Diffraction Analysis: Bruker-Nonius X8 Apex2 Diffractometer; DFT Calculations: Gaussian 09 (B3LYP and DGDZVP).
Data format	*Raw, analyzed.*
Experimental factors	Slow evaporation of CH_2_Cl_2_ solutions of dyes at room temperature gave thin plate-like single crystals that for X-ray analysis
Experimental features	Excited structures determined using single point energy calculations. Vertical electronic excitations of 20 excited states were solved and excited state oxidation potentials were extracted.
Data source location	North Carolina State University, Raleigh, NC, USA.
Data accessibility	Data is with this article. X-ray: Cambridge Crystallographic Data Centre as CCDC 1548989 (https://www.ccdc.cam.ac.uk/structures/Search?Ccdcid=1548989) and CCDC 1548990 (https://www.ccdc.cam.ac.uk/structures/Search?Ccdcid=1548990).
Related research article	Jihye Lim, Malgorzata Szymczyk, Nahid Mehraban, Yi Ding, Lisa Parrillo-Chapman, Ahmed El-Shafei, Harold S. Freeman, Molecular and excited state properties of isomeric scarlet disperse dyes, J. Molec. Struc., Vol. 1161, 254–261.

## Value of the data

• The data illustrate the reliability of current day molecular modeling methods for generating equilibrium geometries of monoazo dyes that are comparable to X-ray crystal structures.
• The data show essential calculations for predicting the molecular and excited state properties of organic dyes.
• The data are useful for further studies on the development of synthetic dyes having high photostability.
• The data show key vertical electronic excitations of 20 excited states for each dye along with the oscillator strength and molecular orbitals involved.

## 1 Data

The data arise from X-ray crystallographic analysis and computational methods in the characterization of isomeric monoazo dyes **Sc2** and **Sc3** for textile fibers. The data are Supplementary material for the study describing the “Molecular and excited state properties of isomeric scarlet disperse dyes” [1].

**Fig. 1:**
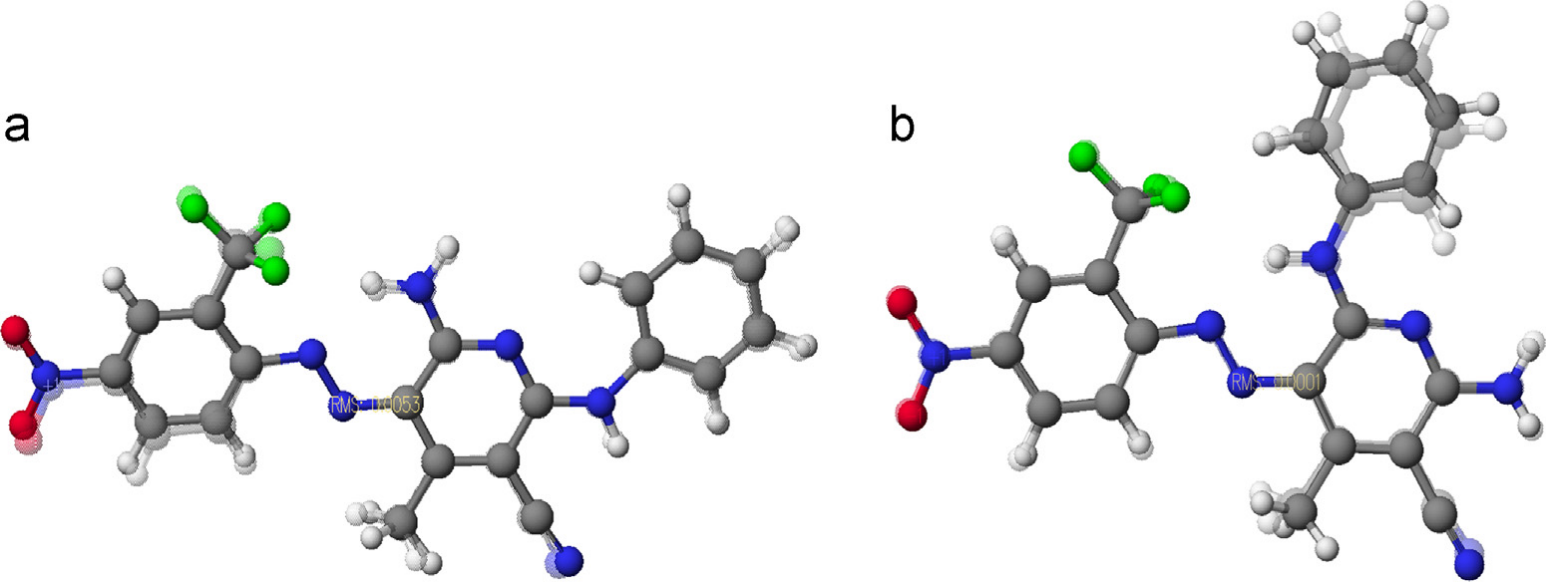
The X-ray structures (top) of **Sc2** (a) and **Sc3** (b) superimposed on the calculated structures (bottom).

The overlay of data from X-ray and computational analysis of dyes **Sc2** and **Sc3** is shown in Fig. 1, to demonstrate the ability of DFT-based calculations to accurately predict the structures of these monoazo dyes. Root-mean squared (RMS) values were 0.0053 for **Sc2** and 0.0001 for Sc3. Other key crystallographic data for the two dyes are summarized in Tables 1 and 2, including the associated crystal systems, space groups, molecular volumes, number of molecules per unit cell, 2*θ*max values, and bond lengths. The latter values are especially helpful in establishing the tautomeric form (azo vs. hydrazone) of the dyes analyzed (cf. N1–N2, N2–C8, N4–C12 data) (Table 3).

**Table 1:** Crystallographic data for the major components of the scarlet disperse dye.

	**Sc2**	**Sc3**
Composition	C_20_H_14_F_3_N_7_O_2_·0.5(CH_2_Cl_2_)	C_20_H_14_F_3_N_7_O_2_
Formula Weight	483.84	441.38
Temperature (K)	100.01	100.04
Crystal system	Monoclinic	Triclinic
Space group	*P*21/*c*	*P*-1
*a* (Å)	20.753(2)	7.5946(2)
*b* (Å)	6.5429(8)	11.0682(3)
*c* (Å)	16.9106(19)	11.3236(3)
*α* (°)	90	80.9900(10)
*β* (°)	113.308(5)	88.6460(10)
*ϒ* (°)	90	81.4210(4)
Volume (Å^3^)	2108.8(4)	929.59(4)
*Z*	4	2
*ρ*calcg (g/cm^3^)	1.524	1.577
*μ* (mm^-1^)	0.243	0.128
*F*(000)	988	452
Crystal dimension (mm)	0.142 × 0.105 × 0.057	0.518 × 0.309 × 0.247
2*θ*max (°)	4.818–46.51	3.642–72.86
Reflections collected	7750	28,451
Independent reflections	2984	9000
Reflections observed	1711	7465
Number of variables	323	299
*R*1 [I > 2*σ*(I)]	0.0542	0.0405
*wR*2 [I > 2σ(I)]	0.1127	0.1152
*wR*1 [all data]	0.1171	0.0506
*wR*2 [all data]	0.1341	0.1241
Largest Diffraction peak/ hole (e^−^/Å^3^)	0.31/− 0.25	0.70/− 0.46
Max. shift in final cycles	< 0.001	< 0.001

**Table 2:** Bond lengths for **SC2**.

**Atom**	**Atom**	**Length/Å**	**Atom**	**Atom**	**Length/Å**
F1	C7	1.341(5)	C2	C7	1.503(5)
F2	C7	1.343(5)	C3	C4	1.379(6)
F3	C7	1.346(4)	C4	C5	1.383(5)
O1	N3	1.226(5)	C5	C6	1.375(5)
O2	N3	1.236(4)	C8	C9	1.419(5)
N1	N2	1.284(4)	C8	C12	1.426(5)
N1	C1	1.419(5)	C9	C10	1.377(5)
N2	C8	1.380(4)	C9	C13	1.499(5)
N3	C4	1.464(5)	C10	C11	1.430(5)
N4	C12	1.344(4)	C10	C14	1.428(5)
N5	C11	1.326(4)	C15	C16	1.389(5)
N5	C12	1.349(4)	C15	C20	1.387(5)
N6	C11	1.358(4)	C16	C17	1.383(6)
N6	C15	1.413(5)	C17	C18	1.377(6)
N7	C14	1.154(5)	C18	C19	1.384(6)
C1	C2	1.400(5)	C19	C20	1.386(5)
C1	C6	1.397(5)	Cl1	C1S	1.730(11)
C2	C3	1.384(5)	Cl2	C1S	1.765(11)

**Table 3:** Bond lengths for **SC3**.

**Atom**	**Atom**	**Length/Å**	**Atom**	**Atom**	**Length/Å**
F1	C7	1.3451(9)	C1	C6	1.4057(10)
F2	C7	1.3380(9)	C2	C3	1.3868(9)
F3	C7	1.3399(8)	C3	C4	1.3838(10)
O1	N3	1.2248(10)	C4	C5	1.3910(10)
O2	N3	1.2243(10)	C5	C6	1.3825(10)
N1	N2	1.2848(8)	C8	C9	1.4176(9)
N1	C1	1.4134(9)	C8	C12	1.4501(9)
N2	C8	1.3675(8)	C9	C10	1.3852(9)
N3	C4	1.4579(9)	C9	C13	1.5007(10)
N4	C11	1.3322(9)	C10	C11	1.4329(10)
N4	C12	1.3351(8)	C10	C14	1.4233(10)
N5	C14	1.1546(9)	C15	C16	1.4013(10)
N6	C11	1.3341(9)	C15	C20	1.3990(10)
N7	C12	1.3503(9)	C16	C17	1.3911(10)
N7	C15	1.4127(9)	C17	C18	1.3868(11)
C7	C2	1.5026(10)	C18	C19	1.3910(11)
C1	C2	1.4084(9)	C19	C20	1.3868(10)

**Fig. 2:**
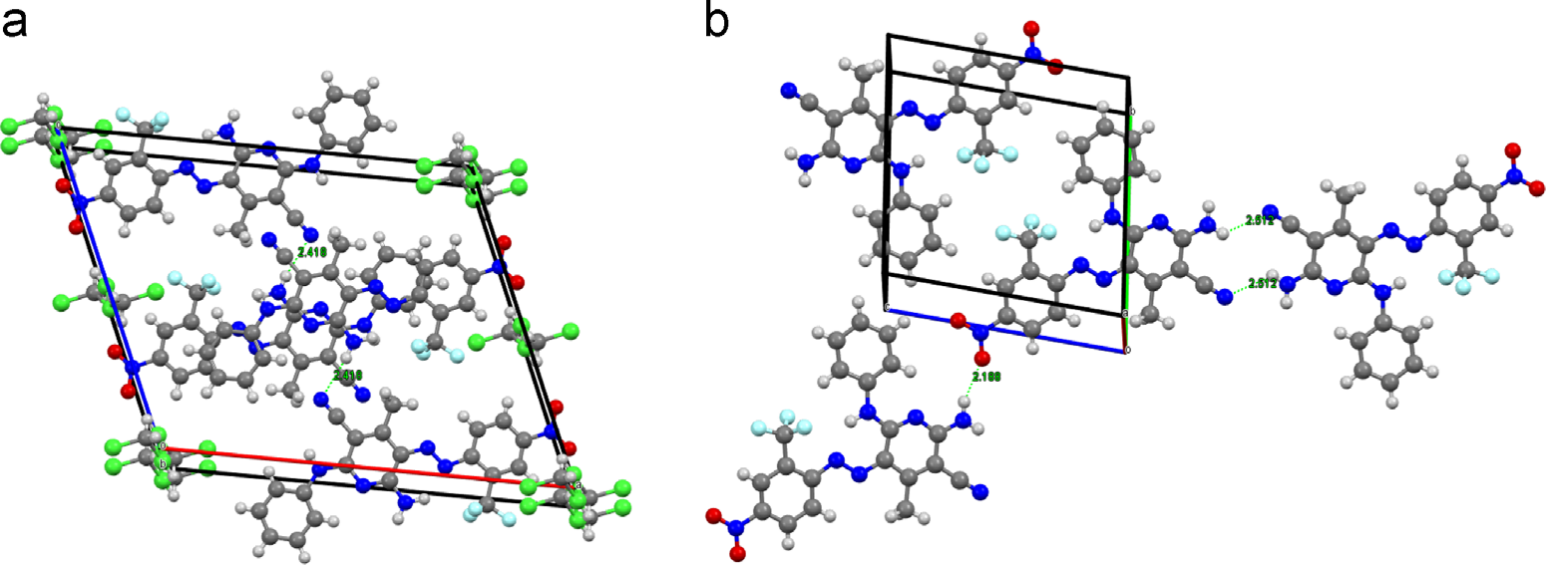
Unit cells showing intermolecular interactions (Å) between molecules of **Sc2** (a) and **Sc3** (b).

Data for intermolecular H-bonding interactions between layers of molecules positioned parallel to each other are given in Fig. 2. The unit cell for **Sc2** shows intermolecular H-bond distances between the NH_2_ and CN groups (2.418 Å). Also seen are short contacts corresponding to intermolecular hydrogen bonds for **Sc3**, namely the NO_2_ and NH_2_ groups (2.188 Å), and the NH_2_ and CN groups (2.512 Å).

Calculation of vertical electronic excitation energies for 20 excited states along with the oscillator strength (f) and molecular orbitals involved for each dye led to the raw data shown in Tables 4 and 5 for **Sc2** and **Sc3**. From these data the excited state oxidation potential (ESOP) for each dye can be extracted.

**Table 4:** Calculated excitation energies and oscillator strengths for the 20 excited states of **Sc2**.

Excited State 1:	Singlet-A	2.3593 eV	525.52 nm	*f* = 1.0963
111 ->114	0.16845			
113 ->114	0.68009			
Excited State 2:	Singlet-A	2.4599 eV	504.02 nm	*f* = 0.0772
111 ->114	0.61550			
111 ->115	− 0.18095			
112 ->114	− 0.19991			
113 ->114	− 0.19321			
Excited State 3:	Singlet-A	3.0046 eV	412.65 nm	*f* = 0.0698
111 ->114	0.22058			
112 ->114	0.66351			
Excited State 4:	Singlet-A	3.3435 eV	370.82 nm	*f* = 0.2245
109 ->114	0.12672			
113 ->115	0.68917			
Excited State 5:	Singlet-A	3.4878 eV	355.48 nm	*f* = 0.0107
110 ->114	0.70043			
Excited State 6:	Singlet-A	3.6637 eV	338.41 nm	*f* = 0.0109
105 ->114	0.14783			
111 ->114	0.18820			
111 ->115	0.60693			
112 ->115	− 0.22758			
Excited State 7:	Singlet-A	3.7276 eV	332.61 nm	*f* = 0.0050
105 ->114	0.59143			
105 ->115	0.31989			
109 ->114	0.10910			
111 ->115	− 0.13494			
Excited State 8:	Singlet-A	3.7570 eV	330.01 nm	*f* = 0.0971
109 ->114	0.65678			
113 ->115	− 0.11278			
Excited State 9:	Singlet-A	3.9326 eV	315.28 nm	*f* = 0.0126
108 ->114	0.54817			
112 ->115	− 0.11815			
113 ->116	0.23227			
113 ->117	− 0.31543			
Excited State 10:	Singlet-A	3.9699 eV	312.31 nm	f = 0.0747
108 ->114	0.12487			
109 ->114	− 0.13850			
111 ->115	0.14977			
112 ->115	0.42170			
113 ->116	0.36207			
113 ->117	0.32511			
Excited State 11:	Singlet-A	4.0260 eV	307.96 nm	*f* = 0.0300
111 ->115	0.17399			
112 ->115	0.47223			
113 ->116	− 0.27249			
113 ->117	− 0.39237			
Excited State 12:	Singlet-A	4.0562 eV	305.67 nm	*f* = 0.0019
107 ->114	0.66738			
107 ->115	− 0.17285			
Excited State 13:	Singlet-A	4.2097 eV	294.52 nm	*f* = 0.0568
108 ->114	− 0.39065			
113 ->116	0.46544			
113 ->117	− 0.34136			
				
Excited State 14:	Singlet-A	4.2568 eV	291.26 nm	*f* = 0.0003
103 ->114	0.62259			
103 ->115	0.32497			
Excited State 15:	Singlet-A	4.4996 eV	275.54 nm	*f* = 0.0045
110 ->115	0.67093			
113 ->119	− 0.15611			
Excited State 16:	Singlet-A	4.5874 eV	270.27 nm	*f* = 0.0100
106 ->114	0.54991			
111 ->116	− 0.24554			
113 ->118	0.31130			
Excited State 17:	Singlet-A	4.5933 eV	269.92 nm	*f* = 0.0026
106 ->114	0.17037			
111 ->116	0.44354			
111 ->117	0.40398			
112 ->116	− 0.20839			
112 ->117	− 0.20558			
Excited State 18:	Singlet-A	4.6084 eV	269.04 nm	*f* = 0.0008
106 ->114	− 0.11476			
111 ->116	− 0.40558			
111 ->117	0.50411			
112 ->116	0.16311			
112 ->117	− 0.15477			
Excited State 19:	Singlet-A	4.6379 eV	267.33 nm	*f* = 0.1619
106 ->114	− 0.30153			
113 ->118	0.60206			
Excited State 20:	Singlet-A	4.7032 eV	263.62 nm	*f* = 0.0287
109 ->115	0.63757			
111 ->116	0.10091			
112 ->117	0.14383

**Table 5:** Calculated excitation energies and oscillator strengths for the 20 excited states of **Sc3**.

Excited State 1:	Singlet-A	2.3700 eV	523.13 nm	*f* = 0.5373
111 ->114	0.11383			
112 ->114	− 0.18480			
113 ->114	0.66822			
Excited State 2:	Singlet-A	2.4473 eV	506.61 nm	f = 0.1416
111 ->114	0.50266			
111 ->115	− 0.16373			
112 ->114	− 0.39149			
113 ->114	− 0.21595			
Excited State 3:	Singlet-A	3.0048 eV	412.61 nm	*f* = 0.4636
111 ->114	0.42774			
112 ->114	0.54660			
Excited State 4:	Singlet-A	3.3508 eV	370.01 nm	*f* = 0.0122
110 ->114	0.64733			
111 ->114	− 0.10057			
113 ->115	0.24591			
Excited State 5:	Singlet-A	3.3624 eV	368.74 nm	*f* = 0.0530
110 ->114	− 0.24661			
113 ->115	0.64269			
Excited State 6:	Singlet-A	3.6418 eV	340.45 nm	*f* = 0.0038
111 ->114	− 0.17595			
111 ->115	− 0.44934			
112 ->115	0.47335			
Excited State 7:	Singlet-A	3.7276 eV	332.61 nm	*f* = 0.0003
105 ->114	0.60898			
105 ->115	0.33006			
Excited State 8:	Singlet-A	3.8726 eV	320.16 nm	*f* = 0.0456
107 ->114	− 0.17843			
109 ->114	0.61937			
111 ->115	− 0.15064			
113 ->116	− 0.15214			
113 ->117	− 0.10277			
Excited State 9:	Singlet-A	3.9310 eV	315.40 nm	*f* = 0.0241
107 ->114	0.39742			
108 ->114	0.35278			
111 ->115	− 0.11320			
112 ->115	− 0.10887			
113 ->116	− 0.27464			
113 ->117	0.30370			
Excited State 10:	Singlet-A	3.9832 eV	311.27 nm	*f* = 0.0557
108 ->114	0.20907			
109 ->114	0.26135			
111 ->115	0.33755			
112 ->115	0.32994			
113 ->116	0.35017			
113 ->117	0.13756			
Excited State 11:	Singlet-A	4.0392 eV	306.95 nm	*f* = 0.5148
107 ->114	0.13790			
111 ->115	− 0.29550			
112 ->115	− 0.32129			
113 ->116	0.49368			
113 ->117	0.12655			
Excited State 12:	Singlet-A	4.1265 eV	300.46 nm	*f* = 0.0008
106 ->114	0.64834			
106 ->115	− 0.17133			
108 ->114	0.12260			
Excited State 13:	Singlet-A	4.1991 eV	295.27 nm	*f* = 0.0884
108 ->114	− 0.40583			
113 ->116	− 0.10829			
113 ->117	0.53076			
Excited State 14:	Singlet-A	4.2585 eV	291.14 nm	*f* = 0.0006
103 ->114	0.62158			
103 ->115	0.32547			
Excited State 15:	Singlet-A	4.2974 eV	288.51 nm	*f* = 0.0161
106 ->114	0.10519			
107 ->114	0.48658			
108 ->114	− 0.34478			
109 ->114	0.10188			
113 ->117	− 0.26919			
Excited State 16:	Singlet-A	4.3887 eV	282.51 nm	*f* = 0.0007
106 ->114	0.10513			
110 ->115	0.65507			
111 ->116	− 0.10839			
112 ->116	0.15738			
Excited State 17:	Singlet-A	4.4058 eV	281.41 nm	*f* = 0.0203
110 ->115	− 0.19596			
111 ->116	− 0.33235			
112 ->116	0.57520			
Excited State 18:	Singlet-A	4.5811 eV	270.64 nm	*f* = 0.0046
111 ->117	− 0.46157			
112 ->117	0.51752			
Excited State 19:	Singlet-A	4.6346 eV	267.52 nm	*f* = 0.1259
111 ->116	0.59135			
112 ->116	0.32627			
Excited State 20:	Singlet-A	4.8246 eV	256.98 nm	*f* = 0.0022
110 ->116	0.43307			
110 ->120	0.13187			
112 ->118	− 0.16383			
113 ->118	0.48759			

## 2 Experimental design, materials, and methods

Single crystal X-ray diffraction analysis was conducted using a Bruker–Nonius X8 Apex2 diffractometer. The frame integration was performed with the program SAINT. The resulting raw data was scaled and absorption corrected using a multi-scan averaging of symmetry equivalent data using SIRPOW [2]. Structures were solved using the program SHELXT [3].

Slow evaporation of CH_2_Cl_2_ solutions of **Sc2** and **Sc3** at room temperature gave thin plate-like single crystals that were suitable for X-ray crystallographic analysis. The equilibrium molecular geometries (EMGs) of **Sc1**, **Sc2** and **Sc3** were calculated in the neutral forms using density functional theory (DFT) employing the generalized gradient approximation (GGA) at the hybrid exchange-correlation energy functional 3-Parameter (Exchange), Lee et al. (B3LYP) [4], [5] and the full-electron basis set Density Gauss double-zeta with polarization functions (DGDZVP) [6], [7], implemented in Gaussian 09. The X-ray structures of **Sc2** and **Sc3** were superimposed on the corresponding calculated molecular geometries and the RMS was calculated in each case. The isosurfaces of the HOMO and LUMO were extracted for each dye from the corresponding checkpoint files. In addition, TD-DFT calculations were performed on the EMGs and the geometry of the excited state structure was calculated using single point energy calculations for each dye. Vertical electronic excitation energies for 20 excited states were calculated for each dye and the excited state oxidation potential (ESOP) for each dye was extracted.
