# Supplementary material for: Data from X-ray crystallographic analysis and DFT calculations on isomeric azo disperse dyes
Source: Data Brief. 2018 Oct 9;21:675–83. doi: 10.1016/j.dib.2018.10.010 (PMC6202689; doi:10.1016/j.dib.2018.10.010)
Supplement: Supplementary file 1 — Supplementary material [file mmc1.docx]

**Conflict of interest**

Declarations of interest: none
